# Rehabilitation from symptomatic drugs overuse: yes, no, may be

**DOI:** 10.1186/1129-2377-16-S1-A34

**Published:** 2015-09-28

**Authors:** Cristina Tassorelli, Grazia Sances, Giorgio Sandrini, Giuseppe Nappi

**Affiliations:** Headache Science Centre, C. Mondino National Neurological Institute, Pavia, Italy; Department of Brain and Behavioural Sciences, University of Pavia, Pavia, Italy

In predisposed subjects with migraine or tension-type headache increasing intake of acute medications is associated with a progressive clinical worsening. When the days of symptomatic drug use reach a given threshold (10 or 15 days/month, depending on the classes of drugs, ICHD-III) and the headache has become chronic for at least three months, a causal relationship is deemed to exist between the medication overuse and the clinical worsening, and the headache is termed medication-overuse headache (MOH). MOH affects nearly 2% of the general population. It represents a highly disabling condition that impacts considerably on the quality of life of sufferers and on the society in general because of high levels of disability and use of healthcare resources.

MOH is treatable: withdrawal from overused drugs leads to a clinically significant improvement in the majority of patients. Even more so, when it is performed within an integrated approach aimed at targeting frequent associated features/conditions (i.e., muscle tenderness and contractures, psychological disturbances, increased stress levels) and in the most appropriate clinical settings (i.e., outpatients clinics for simple MOH, hospitalization for complex MOH). Some Authors also report improvement with prophylactic medication (topiramate, botulinum toxin) alone.

Increasing amount of evidence suggests that the clinical process leading to MOH is paralleled by the establishment of chronic sensitization, which is partly reversed by withdrawal of overused drugs. In addition, several experimental reports confirm that frequent use of analgesics or triptans facilitates nociception, probably via the overexpression of CGRP and neuronal NOS in the trigeminal ganglion.

Clinical practice and analysis of the literature suggest that the beneficial effect of drug withdrawal tends to be more marked and abrupt as compared to the reported slower reduction of headache days when prophylactic medication alone is used. Direct comparison trials are however lacking.

A major issue to be addressed is the high risk of relapse into overuse and pain chronicity following improvement. A good wealth of literature findings has allowed precise quantification of the rate of relapse into overuse and pain chronicity following drug withdrawal (alone or in combination with prophylactic mediation), while data are missing as regards relapse rates following prophylactic treatment alone for MOH.

Taking all these observations into consideration, and awaiting for the necessary evidence from comparative trials, presently the optimal approach to MOH seems to be provided by a multistep process that possibly includes all of the following procedures:

1. Patient's education regarding the need to stop overuse;
2. Withdrawal from overused drugs;
3. Management of comorbid conditions;
4. Optimization of symptomatic medications;
5. Personalization of prophylactic medication.

